# The Genetics of Non-Syndromic Primary Ovarian Insufficiency: A Systematic Review

**DOI:** 10.22074/ijfs.2019.5599

**Published:** 2019-07-14

**Authors:** Roberta Venturella, Valentino De Vivo, Annunziata Carlea, Pietro D’Alessandro, Gabriele Saccone, Bruno Arduino, Francesco Paolo Improda, Daniela Lico, Erika Rania, Carmela De Marco, Giuseppe Viglietto, Fulvio Zullo

**Affiliations:** 1 Department of Obstetrics and Gynaecology, Magna Graecia University of Catanzaro, Catanzaro, Italy; 2 Department of Neuroscience, Reproductive Sciences and Dentistry, School of Medicine, University of Naples Federico II, Naples, Italy; 3 Department of Experimental and Clinical Medicine, Magna Graecia University of Catanzaro, Catanzaro, Italy

**Keywords:** Genetic, Gynecology, Molecular, Precision Medicine

## Abstract

Several causes for primary ovarian insufficiency (POI) have been described, including iatrogenic and environmental factor, viral infections, chronic disease as well as genetic alterations. The aim of this review was to collect all the ge- netic mutations associated with non-syndromic POI. All studies, including gene screening, genome-wide study and as- sessing genetic mutations associated with POI, were included and analyzed in this systematic review. Syndromic POI and chromosomal abnormalities were not evaluated. Single gene perturbations, including genes on the X chromosome (such as *BMP15, PGRMC1* and *FMR1*) and genes on autosomal chromosomes (such as *GDF9, FIGLA, NOBOX, ESR1, FSHR* and *NANOS3*) have a positive correlation with non-syndromic POI. Future strategies include linkage analysis of families with multiple affected members, array comparative genomic hybridization (CGH) for analysis of copy number variations, next generation sequencing technology and genome-wide data analysis. This review showed variability of the genetic factors associated with POI. These findings may help future genetic screening studies on large cohort of women.

## Introduction

Primary ovarian failure (POF) or primary ovarian insufficiency (POI) is defined as primary or secondary amenorrhea in women younger than 40 years of age with follicle stimulating hormone (FSH) .40 IU/L and estradiol levels .50 pg/mL (1-3). The anti-mullerian hormone (AMH) is another indicator of POI risk (2). Recently Venturella et al. (4) described a new methodology to quantify ovarian reserve combining clinical, biochemical and 3D-ultrasonographic parameters called ovAGE.

Several causes for POI have been described, including iatrogenic and environmental factor, viral infections and chronic disease as well as genetic alterations (1, 5). Numerical defects of X chromosome, such as 45,X and 47,XXX, are often associated with ovarian dysgenesis and accelerated follicular atresia (1). Recently, single genes causing non-syndromic POI have been evaluated (6). The aim of this review was to collect all genetic mutations associated with non- syndromic POI. 

## Materials and Methods

Electronic databases were searched from inception of each database, until February 2018 (7). The research was conducted using MEDLINE, EMBASE, Web of Sciences, Scopus, ClinicalTrial.gov, OVID and Cochrane Library as electronic databases. Review of articles also included the abstracts of all references retrieved from the search. We used the following keywords and text words: “Ovarian”, “Failure”, “POF”, “POI”, “Genetic”, “Genomic”, “Syndrome”, “Chromosomal”, “Premature”, “Primary” and “Infertility.” 

All studies assessing genetic mutations associated with non-syndromic POI, including mutations located in X and autosomal chromosomes, were analyzed. Syndromic POI and chromosomal abnormalities (e.g. numerical defects, X-structural abnormalities, X-autosome translocations and autosomal rearrangements) were not evaluated. Pleiotropic single gene disorders (e.g. Fragile X syndrome), mitochondrial gene diseases (e.g. Perrault syndrome), and multiple malformation syndromes (e.g. cerebellar ataxia) were also excluded.

## Results

### Single genes causing non-syndromic primary ovarian insufficiency

Many genes whose product is known playing a role in human folliculogenesis (candidate genes) have been studied (6).

### Genes on the X chromosome

#### *Bone morphogenetic protein 15 (BMP15)* (Xp 11.2)

BMP15 is a member of the transforming growth factor (TGF) family involved in stimulating folliculogenesis. It promotes follicle maturation by regulating granulosa cell differentiation and proliferation (8). Evidences from animal models primarily suggested the possible involvement of BMP15 in pathogenesis of POI. Bmp15 knockout female mice presented subfertility and defective ovulation processes (9). Concerning human disease, the first evidence was reported in the 2004 (10). They identified a heterozygous p.Y235C missense mutation in two POI patients, caused by decrease of granulosa cell proliferation through a dominant negative effect. Many variants in *BMP15* gene have been described in Caucasian, Indian and Chinese patients with POI (10-17). These variants in *BMP15* lead to impaired dimerization, reducing production of mature BMP15 active protein and subsequent defective granulosa cell signaling, in addition to increased follicle atresia.

#### *Progesterone receptor membrane component I (PGRMC1)* (Xq22-q24)

PGRMC1 is a putative progesterone-binding membrane receptor, expressed in various tissues (18-22). Authors (21) have identified a mother and daughter with POI carrying an X-autosome translocation [t(X;11)(q24;q13)] and a sporadic missense mutation (p.H165R), in the cytochrome b5 binding domain of *PGRMC1*. These variants are associated with lower levels of PGRMC1, and consequently ovarian cells apoptosis. Wang et al. (23) catalogued a different missense mutation (c.556C.T; p. P186S), however, more research is needed to establish association of this variation with POI.

#### *Androgen receptor (AR)* (Xq12)

The *AR* gene is related with reproduction, as well as sex differentiation. AR is present in ovary, precisely in granulosa cells, and it is useful to follicles development. Shiina et al. (24) found that deficiency of AR in female mice may lead to a POI-like phenotype and dysregulation of many important genes involved in folliculogenesis. These results probably indicate that regular folliculogenesis need AR-mediated androgen action. We reported two examples of mutations on AR gene linked with POI: CAG repeat length in exon 1 and two missense mutations (p.T650A and p.O658K) (25-30).

#### *Premature ovarian failure, IB (POFIB)* (Xq21.2)

POFIB is considered as a region codified by OMIM, located within the critical POI1 region. In a patient with secondary amenorrhea (POI), this region was interrupted by a breakpoint in an X-autosome translocation. Lacombe et al. (31) proved linkage to Xq21 in a family having five patients with POI. A homozygous p.R329Q mutation was identified, leading to decreased ability to bind actin filaments.

#### *Dachshund family transcription factor 2 (DACH2)* (Xq21.3)

*DACH2* is located on Xq21.3 and involved in POI (32) with two missense mutations, p.R37L and p.F316S (33). 

#### *Fragile X mental retardation I (FMRI)* (Xq27.3)

*FMR1* is an X-linked gene located at Xq27.3 and characterized by CGG repeats in its 5’ untranslated region. Full mutation, consisting of >200 CGG repeats, is associated with the fragile X-syndrome in male carriers, but not with POI. CGG repeats ranging from 55 to 199 are known as *FMR1* premutation and recognized as common gene involved in POI. *FMR1* premutations have been identified more frequently in POI patients having a positive family, compared to sporadic forms (34). *FMR1* premutations are more frequently identified in Caucasian patients with POI than general population (35). Compared to Caucasian population, the prevalence of *FMR1* premutations is lower in Asian POI patients (36). Some studies indicate that there is no association between Intermediate range of CGG repeats (41or 45-54 repeats) and POI (37). FMR1 protein is a RNA binding protein highly expressed in fetal ovary germ cells and granulosa cell. *FMR1* premutations associate with decreased size of initial follicular pool (38-40).

### Genes on autosomal chromosomes

#### *Growth differentiation factor 9 (GDF9)* (5q31.1)

As with *BMP15, GDF9* is part of TGF gene family presented in oocytes. These characteristics make it an important candidate gene for POI. New heterozygous variants have been found in European, Caucasian and Asian patients (41-43), but not in Japanese and New Zealand population (44, 45). Norling et al. (46) performed high-resolution array comparative genomic hybridization (CGH) in 26 POI Swedish cases and discovered a partial GDF9 gene duplication with 475 bp length.

#### *Folliculogenesis specific bHLH transcription factor (FIGLA)* (2p13.3)

*FIGLA*, encodes an oocyte- specific, basic helix-loop- helix (bHLH) transcription factor, which is necessary for the first stage of folliculogenesis. It regulates expression of zona pellucida genes. Three variants have been described, by Zhao et al. (47), in 100 Chinese patients with POI, including missense mutation in two women, deletion of p.G6fsX66 in one woman and deletion p.140delN in another woman. The Deletion of p.G6fsX66 may cause POI through a mechanism of haploinsufficiency, while the deletion of p.140delN may induce impaired heterodimerization. Tosh et al. (48) also identified an intronic variant in 209 Indian patients with POI. 

#### *New ovary homeobox gene (NOBOX)* (7q35)

*NOBOX* is an ovary homeobox gene involved in first stages of folliculogenesis. Rajkovic et al. (49) identified NOBOX role, using knockout mouse models. In female mice, they determined fibrous tissues replacing follicles, causing similar phenotype to non-syndromic ovarian failure. Lechowska et al. (50) identified that NOBOX deficiency may lead to POI through a disorder caused due to communication between somatic cells and oocytes during embryonic development. This results in anomalous junctions between joined oocytes within syncytial follicles. Several NOBOX mutations have been detected in Caucasian POI patients (51-53). For instance, p.R355H mutation associates with a decrease in *NOBOX* DNA binding activity and a dominant negative effect. No *NOBOX* variant has been found in Chinese women with POI (54).

#### *Nuclear receptor subfamily 5, group A, member I (NR5AI): Steroidogenic factor1 (SF1)* (9q33)

*NR5A1, SF1,* is a nuclear receptor involved in early gonadal differentiation. NR5A1 modulates the transcription of genes implicated in steroidogenesis such as AMH, nuclear receptor subfamily 0, group B, member 1 (DAX1), CYP11A, steroidogenic acute regulatory protein (StAR), CYP17A1, CYP19A1 and INHA. *N5RA1* knockout in mouse granulosa cells induced hypoplastic ovaries, reduced number of oocytes and infertility. *NR5A1* mutations have been identified by Lourenco et al. (55) in four families with history of POI and in 2/25 women with sporadic POI. These mutations, not identified in control patients, were associated with altered transactivation activity of factors involved in follicle growth. In a study including 356 Dutch patients with POI, nine different mutations were determined in coding regions of *NR5A1* (56). Jiao et al have identified Py5D mutation, as a non-domain region, in Chinese women with POI.

#### *Estrogen receptor 1 (ESR1)* (6q25.1)

*ESR1* gene is one of the two estrogen receptor subtypes. It has been considered as a potential candidate gene for POI (57). ESR1 knockout mice models showed an early loss of fertility due to the impaired follicles maturation. Qin et al. (58) analyzed 41 single nucleotide polymorphisms (SNPs) in 400 cases and 800 women controls. They found one SNPs related to POI in *ESR1* (rs2234693) in Korean and Dutch women. They also identified two novel SNPs in HK3 and BRSK1 (rs2278493 and rs12611091, respectively), probably involved in POI pathogenesis.

#### *FSH receptor* (FSHR) (2p21-p16)

FSH/FSHR signaling has an important role in regular gonadal function. A study, composed of 75 patients with hypergonadotrophic ovarian dysgenesis and primary or secondary amenorrhea cases, discovered homozygous mutations (59-61). These mutations are common (0.96%) in Finnish women, but rare in the other populations (13, 62-70).

#### *TGF, beta receptor III (TGFBR3)* (1p33-p32)

Human *TGFBR3* is located at 1p33-p32 and translates the TGF-beta type III receptor. In Chinese women with idiopathic POI, two missense variants, p.E459G and p.P825L were identified. The third one, p.P775S, was discovered in an Indian POI case.

#### *G protein-coupled receptor 3 (GPR3)* (1p36.1-p35)

*GPR3* gene is an element of the G protein coupled receptor family. In Gpr32/2 mice, higher quantity of the oocytes, in antral follicles, quickly restarted meiosis. Similar variant (c.135G.A; p.V45V) was discovered in one Chinese woman. 

#### *Wingless-type MMTV integration site family, member 4 (WNT4)* (1p36.23-p35.1)

*WNT4* translates a secreted extracellular signaling protein which plays a role in female sex differentiation. 

#### *Inhibins (INH): Inhibin, alpha (INHA) (2q35), Inhibin, beta A (INHBA)* (7p15-p13), Inhibin, beta B (INHBB) (2cen-q13)

INH is a member of TGF-b family. In New Zealand (7%), Indian (11.2%) and Italian (4.5%) women with POI a common missense variation c.769G.A (p.A257T) was found in the *INHA* gene. Regarding *INHBA*, new missense variations were found only in Indian POI women. Causative variation in *INHBA* and *INHBB* genes has not been found yet.

#### *POU class 5 homeobox 1 (POU5FI)* (6p21.31)

In *NOBOX* gene knockout mice, *POU5F1* reproduction factor gene is significantly down-regulated, making it a possible aspirant gene for POI. A study found one non- synonymous mutation (p.P13T) in 175 Chinese women with POI.

#### *Class 5 homeobox MutS homolog 4 (MSH4)* (1p31) and *MSH5* (6p21.3)

*MSH4* (1p31) and *MSH5* (6p21.3) are members of mammalian DNA mismatch repair genes family. Mouse model carrying an inactivating *MSH4* mutation in the germ line, identified a severe disruption of meiosis in *Msh4* mutant females and males. Furthermore, failure of the chromosome pairing in oocytes caused apoptosis increase, loss of oocyte pool and ovarian structures. A heterozygous mutation p.P29S in the MSH4-binding domain of *MSH5* in Caucasian patients. Guo et al. (36), using whole exome sequencing in a Chinese pedigree with POI, identified a homozygous missense mutation (p.D487Y) in the *MSH5* gene of two sisters with POI. In addition, POI phenotype was determined in mice carrying the homologous mutation.

#### *Cbp/p300-interacting transactivator, with Glu/Asp-rich carboxy- terminal domain, 2 (CITED2)* (6q23.3)

*CITED2* gene encodes a protein inhibiting transactivation of HIF1A-induced genes. *CITED2* mutations may lead to idiopathic POF. Fonseca et al. (30) analyzed 139 patients with POI and 290 controls. They identified a missense mutation p.P202T in *CITED2* of one case. More studies are needed to identify the role of *CITED2* mutations in POI pathogenesis.

#### *Spermatogenesis and oogenesis specific basic helix-loop-helix transcription factor I (SOHLHI)* (9q31.3) and *SOHLH2* (13q13.3)

*SOHLH1* and *SOHLH2* are only expressed in primordial follicles and they encode testis-specific transcription factors. They are needed for spermatogenesis, oogenesis and early folliculogenesis. Sohlh2 knockout adult female mice are infertile, since differentiation of the oocyte during early oogenesis is compromised and oocytes are quickly lost. 

#### *Phosphatase and tensin homolog (PTEN)* (10q23.3)

*PTEN* gene, located on chromosome 10q23.3, encodes protein which negatively regulates intracellular levels of PI3K and consequently AKT/PKB signaling pathway in cells. It has been found essential maintenance of dormancy of primordial follicles. In mice with PTEN deficiency, the entire pool of primordial follicles was prematurely activated and early depleted. 

#### Nanos homolog 1, 2, 3 (Drosophilia): *NANOSI* (10q23.11), *NANOS2* (19q13.32), *NANOS3* (19q13.13)

*NANOS* gene belongs to a family needed for primordial germ cell (PGC) evolution and conservation. Three homologs of this molecule have thus far been determined: *NANOS1, NANOS2* and *NANOS3. NANOS2* defect solely cause spermatogonia failure, while defective PGC conservation in both males and females with NANOS3 deficiency was observed. *NANOS3* variants have been studied in 80 Chinese and 88 Caucasian POI patients. No causative variant was detected in coding exons, while a different study found a hypothetically important heterozygous mutation (c.457C.T; p.R153W). In addition, new homozygous variation (c.358G.A; p.E120K) was discovered in two sisters with POI. 

#### *Cyclin-dependent kinas inhibitor IB (CDKNIB)* (12p13.1-p12)

CDKN1B, also known as P27 or KIP1, translates an inhibitor involved in growth and differentiation of several tissue. It is responsible for follicle atresia. Authors, showed early follicle depletion in *CDKN1B* knockout mice. 

#### *Anti-mullerian hormone receptor, type II (AMHR2)* (12q13)

*AMHR2* translates an AMH receptor. It has a central part in the conservation and growth of reproductive organs in humans (6). Studies found a polymorphism (c.-482 A.G; rs2002555) in *AMHR2* of some populations, but not in the others (58). Recently, two new missense variations in a group of Chinese women with POI was noted by Qin et al. (58).

#### *Forkhead box protein L2 (FOXL2)* (3q23)

*FOXL2* belongs to forkhead family of transcription factors, expressed in mammalian undifferentiated granulosa cells. It may play a role in fertility conservation. *FOXL2* knockout mouse models presented an early activation and decrease of primordial follicles. Moreover, one study recently identified that *FOXL2* is essential for regulation of AMH. Three *FOXL2* mutations have been found in almost 5% of non-syndromic POI patients, including c.772T>A (p. Tyr258Asp), c.661_690del (p.Ala221_Ala230del) and c.560G>A (p.Gly187Asp). 

#### *Forkhead box 03 (FOX03)* (6q21)

*FOXO3* gene, located at 6q21, belongs to the forkhead family of transcription factors. FOXO3 is involved in oocyte quiescence. It suppresses initiation of follicular maturation and controls the rate of utilization of the reproductive potential. FOXO3 null mice presented an early global activation of primordial follicles, until a premature depletion of the primordial oocyte pool. Several *FOXO3* variants have been identified in different ethnic groups with different frequency.

#### *Forkhead box 01 (FOX01)* (13q14.1)

*FOXO1*, belonging to forkhead family of transcription factors, may be involved in follicular steroidogenesis and plays a role in follicle development by controlling granulosa cell proliferation, apoptosis and differentiation. In 60 patients with POI from New Zealand and Slovenia two variants have been found. These variants included one missense mutation, and one 5’ UTR mutation (p.P84L and c.-30C.T, respectively).

#### *The Wilms tumor 1 (WT1) gene *(11p13)

*WT1* gene is translated to a transcription factor expressed in granulosa cells. Variations of this gene lead to defects in granulosa cell polarity, which could explain the origin of POI and POI phenotype in knockout mice.

#### *The Splicing Factor 1 (SF1) gene* (11q13.1)

SF1 has a significant role in ovarian development and it appears to cause POI in Tunisian women by reducing estradiol levels.

#### *Spalt-like transcription factor 4 (SALL4)* (20q13.2)

*SALL4* encodes a zinc finger transcription factor which plays role in the developing limbs and motor neurons. *SALL4* might also be involved in conferring totipotency on oocytes. So, 100 Han Chinese women with non-syndromic ovarian failure were screened and two variants were identified in this gene, including p.Val181Met and p.Thr817Ala. 

#### *Meiotic protein covalently bound to DSB (SP011)* (20q13.31)

*SPO11* encodes a protein which is essential for formation of double stranded breaks (DSBs) (or initiation of meiotic DSBs). It is also required for the chromosome segregation. Infertility has been observed in Spo11 knockout mice due to impaired meiosis and depletion of oocytes. 

#### *DNA meiotic recombinase I (DMCI)* (22q13.1)

*DMC1* encodes a member of the recombinases family (also called DNA strand-exchange proteins). Recombinases are essential in repairing breaks of double-strand DNA. 

### Genome-wide studies in primary ovarian insufficiency

The candidate gene approach and cytogenetic studies have provided some important results so far. Recently, new strategies have been performed for identifying new genes and unknown pathways associated with POI development. These strategies include linkage analysis in families with multiple affected patients, array-CGH for analysis of copy number variants (CNVs), genome-wide association (GWA) studies (GWAS), genome-wide sequencing of exomes (WES) and the whole genome sequencing (WGS) as well as the next generation sequencing (NGS) (6).

### Genome-wide association studies

In genetics, a GWAS consists of the analysis of a genome- wide set of genetic variants to discover their association with a trait. Especially, GWASs focus on SNPs. GWA studies use high-throughput genotyping technologies to investigate the entire genome and common SNPs assay, without any prior hypotheses regarding the mechanism or biological pathways. Then, this analysis consists of studying SNPs in affected and control women. 

Thanking GWAS, several potentially POI related loci were identified in Chinese, Korean, and Dutch women, but no interesting finding was confirmed by replicating these studies. The major limitation of GWAS is lack of statistical power, due to population proportions and sample size. POI is actually a rare disease, so it could be difficult to increase the sample size.

### Genome-wide association studies based family linkage analysis 

Some GWAS studies also showed a dominant pattern of inheritance. A large consanguineous Middle-Eastern family with POI, presenting an autosomal recessive pattern of inheritance, was subject of GWA. They identified two regions including 7p21.1-p15.3 and 7q21.3-q22.2.

### Genome-wide association studies based on age of menopause

A new strategy to identify genetic mechanism involved in POI might consist of using evidences from shared genetic susceptibility natural menopause or early menopause women. Studies have identified a significant association between POI and three SNPs including rs2278493 in hexokinase 3 (*HK3*), rs2234693 in estrogen receptor 1 (ESR1) and rs12611091 in BR serine/threonine kinase 1 (*BRSK1*) (6).

### Copy number variants

CNVs are a structural variants involving DNA regions >1 kb. They consist of alterations in the copy number of specific regions such as deletions and duplications. They can be either inherited or spontaneously arisen de novo, leading to phenotypic variations and disease. Recently, array-CGH has been used to search CNVs potentially involved in POI. Studies have identified eight statistically significant different CNVs in chromosomal regions (1p21.1, 5p14.3, 5q13.2, 6p25.3, 14q32.33, 16p11.2, 17q12 and Xq28) of five genes playing role in reproduction, includingDynein axonemal heavy chain 5 (*DNAH5*), NLR family apoptosis inhibitory protein (*NAIP*), dual specificity phosphatase 22 (*DUSP22*), nuclear protein 1 transcriptional regulator (*NUPR1*) and AKT serine/threonine kinase 1 (*AKT1*). 

Ledig et al. (13) performed array-CGH analysis in 74 German patients with POI and identified 44 rearrangements (deletions and insertions) through several genes involved in meiosis, DNA repair and folliculogenesis. Seventeen novel microduplications and seven novel microdeletions, six of which were located in the coding regions of 8q24.13, 10p15-p14, 10q23.31, 10q26.3, 15q25.2 and 18q21.32 have been identified. Two novel microdeletions were discovered to cause haploinsufficiency for *SYCE1* and *CPEB1* genes playing a role in ovarian failure in knockout mouse models. In 2014, Norling et al. (46) performed a case-control study. They used arrayCGH to identify CNVs in 26 POI patients. Eleven unique CNVs were found in 11 patients, including a tandem duplication in part of the *GDF9* gene promoter region, known as probable causative gene for POI. Further studies in much larger POI samples are needed to identify novel CNVs and to discern the utility of array-CGH in replacing conventional karyotyping.

### Whole exome sequencing

WES allows simultaneous analysis of base pairs across the entire exome. This was traditionally defined as the sequence encompassing all exons of protein coding genes in the genome. Six WES was performed in non-syndromic inherited POI. It has been indicated that majority of the candidate genes play role in meiosis and DNA repair, in this study (Table 1).

**Table 1:** Identified genes using WES associated with non-syndromic inherited POI


Abbreviation	Genes

*STAG3*	*Stromal antigen 3*
*SYCE1*	*Synaptonemal complex central element*
*eIF4ENIF1*	*Eukaryotic translation initiation factor 4E nuclear import factor*
*CLPP*	*Caseinolytic mitochondrial matrix peptidase proteolytic subunit*
*C10ORF2*	*Chromosome 10 open reading frame 2*
*HFM1*	*ATP-dependent DNA helicase homolog*
*MCM8* and *MCM9*	*Minichromosome maintenance complex component 8 and 9*
*LARS2*	*Leucyl-tRNA synthetase 2, mitochondria; C10orf2*
*HSD17B4*	*Hydroxysteroid (17-beta) dehydrogenase 4*


WES; Whole exome sequencing and POI; Primary Ovarian Insufficiency.

### Whole genome sequencing and next generation sequencing

NGS has revolutionized genomic research. Thanks to NGS an entire human genome can be sequenced in a single day and new molecular players in POI can be identified. Fonseca et al. (30) performed a retrospective case-control cohort study including 12 patients with non-syndromic POI and 176 controls. NGS was used to sequence complete coding regions of 70 candidate genes in POI patients and mutations in *ADAMT19, BMPR2* and *LHCG* were identified. 

## Discussion

This review includes almost all genetic abnormalities and genes linked with non-syndromic POI. This exhibits the importance and variability of genetic elements involved in POI genesis and identified by different techniques. Different conclusion can be made based on our review. First, several genes come out as POI candidates, but only a little part of them have been established unequivocally causative factor, by functional tests. Second, remarkable differences in frequency exist among different ethnic groups. New studies with a large sample sizes should more imply disparate ethnic groups. Moreover, interactions between gene-gene and protein-protein are not yet entirely clear. Recent and future advances in sequencing techniques will help find other novel genes involved in POI. Finally, discovering the pathogenesis and molecular bases of POI is useful not only to understand the ovarian physiology, but also to improve genetic and fertility counseling. Once new variants are found, they can help prognosticate the age of menopause. In future, findings from this review may help large genetic screening studies on infertility and may help women plan their fertility.

## Conclusion

This review showed variability of the genetic factors associated with POI. These findings may help future genetic screening studies on large cohort of women.
